# Breast cancer screening among Hispanic and non‐Hispanic White women by birthplace in the Sister Study

**DOI:** 10.1002/cam4.4563

**Published:** 2022-02-12

**Authors:** Charlotte J. Talham, Francisco A. Montiel Ishino, Katie M. O'Brien, Dale P. Sandler, Faustine Williams

**Affiliations:** ^1^ Division of Intramural Research National Institute on Minority Health and Health Disparities National Institutes of Health Bethesda Maryland USA; ^2^ Epidemiology Branch National Institute of Environmental Health Sciences, National Institutes of Health, Research Triangle Park North USA

**Keywords:** breast cancer, epidemiology and prevention, screening, Women's cancer

## Abstract

**Background:**

Hispanic/Latina women are less likely to be diagnosed with local stage breast cancer than White women. Additionally, foreign‐born women have lower mammography rates than US‐born women. We evaluated the combined effect of birthplace and race/ethnicity on screening habits of women at higher‐than‐average risk of breast cancer.

**Methods:**

Multinomial logistic regression was used to evaluate breast cancer screening in 44,524 women in the Sister Study cohort. Screening methods ascertained at enrollment (2003–2009) included mammography, ultrasound, and magnetic resonance imaging. Timing of screening was assessed as *recently* (≤2 years ago)*, formerly* (>2 years ago), and *never* screened. Adjustments included sociodemographic, socioeconomic, and health variables.

**Results:**

Most women in the sample were US‐born non‐Hispanic/Latina White (92%), were ≥50 years old (73%), had one first‐degree female relative with breast cancer (73%), and were screened in the past two years (97%). US‐born Hispanic/Latina women had higher odds (odds ratio [OR] = 1.47, 95% confidence interval [CI] = 1.08–2.00) than US‐born non‐Hispanic/Latina White women of not having received a breast cancer screening in the past 2 years, relative to a recent screening. Similarly, foreign‐born Hispanic/Latina women had higher odds (OR = 1.63, 95% CI = 1.10–2.41) than US‐born non‐Hispanic/Latina White women of never having received a breast cancer screening.

**Conclusion:**

We observed that Hispanic/Latina women have higher odds of never and dated breast cancer screenings compared to US‐born White women. Birthplace and race/ethnicity each contribute to disparities in who receives preventative health care in the United States. It is critical to include birthplace when evaluating health behaviors in minority groups.

## Graphical abstract

Multinomial logistic regression was conducted on 44,524 Hispanic and non‐Hispanic White women from the Sister Study cohort to evaluate the associations between combined birthplace and race/ethnicity and timing of most recent breast cancer screening, while controlling for sociodemographic, socioeconomic, and health factors. Foreign‐born Hispanic women were found to have higher odds of never receiving a breast cancer screening compared to US‐born non‐Hispanic White women. Similarly, US‐born Hispanic women were found to have higher odds of having an outdated breast screening compared to US‐born non‐Hispanic White women.

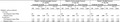

## 1 INTRODUCTION

Breast cancer is the leading cause of new cancer diagnosis and the second leading cause of cancer death among women in the United States.^1^ Among Hispanic/Latina women, however, breast cancer is the primary cause of cancer death in the United States.^2^ Despite an overall decline in breast cancer mortality since 1989, stark racial and ethnic disparities persist in breast cancer diagnosis, treatment, and survival.^3^

Women with a family history of breast cancer are at higher‐than‐average risk of developing the disease. Relative to women with no family history, breast cancer risk is 1.8 times higher in women with one first‐degree relative and 2.93 times higher in women with two first‐degree relatives with the disease.^4^ The risk further increases in women whose family members were young (aged <50) at the time of diagnosis.^5^ Outside of family history, important risk factors for breast cancer include behavioral health factors such as alcohol consumption,^6^ physical inactivity,^6^ and, for postmenopausal women, high body mass index (BMI).^7^ Therefore, adherence to cancer preventive lifestyles including screening recommendations is especially important among women at heightened risk due to family history.^8^

Breast cancer screening is an important preventive health measure used to detect breast cancer in an early, easy‐to‐treat stage. Early detection is associated with more successful treatment and higher rates of survival in women.^1^ The American Cancer Society (ACS)^1^ recommends that women at high risk of breast cancer begin annual breast magnetic resonance imaging (MRI), in addition to mammography screening, at age 30. On the other hand, the United States Preventative Services Task Force (USPSTF) recommends biennial screening between the ages of 50 and 74 for women at average risk and states that women with breast cancer family history may benefit from initiating screening in their 40s. However, prior to altering their breast cancer screening guidelines in 2009, USPSTF recommended shorter screening intervals and earlier initiation of screening. The previous recommendation advised mammography screening every 1–2 years for average‐risk women beginning at age 40.^9^ Minority populations, however, may face numerous obstacles to adhering to screening guidelines.

The United States is currently home to around 44.8 million immigrants, 44.3% of whom are Hispanic/Latino and over half of whom are female.^10^ The process of migration can be highly disruptive to women's lifestyles, health behaviors, and care‐seeking practices.^11^ Once in the United States, foreign‐born and immigrant women face financial, linguistic, cultural, and logistical barriers to seeking and affording care, including breast cancer screenings.^11^, ^12^, ^13^ Additionally, acculturation, the process through which individuals adopt the societal norms, values, and practices of a new host culture, can lead to both positive and negative effects on health.^14^, ^15^ Immigrants to the United States generally engage in unhealthy lifestyle behaviors at lower rates than their native‐born counterparts.^16^ Despite this, the process of acculturation coincides with lifestyle changes over time, including changes in preventive health behaviors, which frequently converge to host culture levels.^16^ Research by Abraído‐Lanza *et al*.^14^ found that among Hispanics/Latinos, acculturation is associated with greater likelihoods of alcohol consumption, smoking, and high BMI. On the other hand, Clarke *et al*.^17^ found that length of time in the United States, a commonly used proxy for acculturation, is positively associated with more breast cancer screening.

In the United States, Hispanic/Latino individuals experience lower rates of many cancers than non‐Hispanic/Latino White individuals, including female breast cancer.^2^ However, while breast cancer incidence among Hispanic/Latina women is 29% lower than among non‐Hispanic/Latina White women, Hispanic/Latina women are less likely to be diagnosed with breast cancer at a local stage, even when accounting for age and socioeconomic status.^2^ Between 2011 and 2015, 57% of breast cancers in Hispanic/Latina women were diagnosed at a local stage compared to 65% in non‐Hispanic/Latina White women.^2^ One reason for this disparity is that Hispanic/Latina women have lower mammography utilization prevalence than non‐Hispanic/Latina White women, although the gap has narrowed in recent years.^2^ Healthy People reported that in 2018 adherence to USPSTF guidelines among Hispanic/Latina women was 71.5% compared to 73% among non‐Hispanic/Latina White women.^18^ However, mammography utilization varies by subpopulation with prevalence as low as 51% in Cubans.^2^ Additionally, adherence to screening guidelines is higher among US‐born Hispanic/Latina women than among their foreign‐born counterparts.^19^, ^20^

Given the rapidly growing US Hispanic/Latino population, the known health risks associated with acculturation, and the benefits of adherence to screening guidelines, it is essential to understand the disparities in breast cancer screening utilization observed in the United States. This need is even more pronounced among women with family history of breast cancer due to their heightened risk. Efforts to increase screening and reduce stage‐of‐diagnosis disparities among Hispanic/Latina women in the United States may be more effective if we better understand the composite effects of birthplace and race/ethnicity on breast cancer screening. To address this need, we analyzed the Sister Study cohort baseline data to investigate the associations between combined birthplace and race/ethnicity and timing of most recent screening, while accounting for important risk factors such as family history and behavioral exposures. Our study contributes to the breast cancer disparity literature as one of the few to use national cohort data to assess screening use by birthplace/race/ethnicity among a higher‐than‐average‐risk population.

## 2 METHODS

### 2.1 Data source

We used data collected by the Sister Study, a longitudinal prospective cohort study of 50,884 women residing in the United States, including Puerto Rico. Women were eligible for enrollment if they were between the ages of 35 and 74 and had a full‐ or half‐sister who had been diagnosed with breast cancer but had never been diagnosed with breast cancer themselves. Participants provided written informed consent at the time of enrollment (2003–2009). A detailed description of the Sister Study design and methods can be found at https://sisterstudy.niehs.nih.gov and in past publication.^21^ The Sister Study was approved by the Institutional Review Board (IRB) of the National Institute of Environmental Health Sciences and the Copernicus Group IRB. The present analysis included Sister Study cohort baseline data from data release 7.2. Data are restricted but available via application to the Sister Study website.^22^

Due to small numbers, women who identified as foreign‐born non‐Hispanic/Latina and either Black, American Indian/Alaskan Native, Asian, and Native Hawaiian or other Pacific Islander were excluded. Therefore, after omitting *n* = 2 women due to study withdrawal, we restricted our analysis to 44,815 women who identified as non‐Hispanic/Latina White or Hispanic/Latina. We further excluded women due to incomplete breast cancer screening information (*n* = 8), prophylactic mastectomy (*n* = 228), and previous diagnosis of invasive breast cancer or ductal carcinoma in situ or otherwise ambiguous diagnosis information (*n* = 55). Therefore, information from 44,524 women contributed to this analysis.

### 2.2 Outcome of interest

The outcome of interest in our analysis was timing of most recent breast cancer screening. We considered mammography, ultrasound, and MRI as forms of breast cancer screening. The timing of participants' most recent mammogram was assessed by the questions, “Have you ever had a mammogram? [yes, no],”, and if yes, “Was your last mammogram… [less than a year ago, one to two years ago, more than two years ago].”. Participants also received questions about other forms of screening including, “Have you ever had a screening ultrasound of the breast? [yes, no]” and “Have you ever had a screening MRI of the breast?”. Although participants were not asked about the timing of their most recent ultrasound or MRI, we assumed women who had received one of these forms of screening had transitioned from mammography to specialized screening methods due to higher‐than‐average risk. Our outcome variable, timing of most recent breast cancer screening, had three levels: (1) *recently screened*, which consisted of women who had received a mammogram in the past two years and/or received an MRI or ultrasound; (2) *formerly screened*, which consisted of women who had received a mammogram over two years ago; and (3) *never screened*, which consisted of women who had never received any of the aforementioned breast cancer screening types.

### 2.3 Independent variables

#### 2.3.1 Sociodemographic characteristics

Sociodemographic factors included combined race/ethnicity and birthplace, age, and marital status. This analysis was restricted to women who identified as non‐Hispanic/Latina White and Hispanic/Latina. Racial/ethnic identity was assessed by the questions, “Do you consider yourself to be Hispanic or Latina? [yes, no]” and “What race do you consider yourself to be? You may choose one or more of the following: [American Indian or Alaskan Native, Asian, Black or African American, Native Hawaiian or other Pacific Islander, White].” Women who responded “yes” to the former question were considered Hispanic/Latina and women who responded “no” to the former and only “White” to the latter were considered non‐Hispanic/Latina White. Foreign birth was considered nativity in a US territory, commonwealth, or outside of the United States. Combined birthplace and race/ethnicity thus had four categories: (1) US‐born non‐Hispanic/Latina White; (2) foreign‐born non‐Hispanic/Latina White; (3) US‐born Hispanic/Latina; and (4) foreign‐born Hispanic/Latina. Age was divided into three categories: (1) under 40; (2) 40–49; and (3) 50 and older. Finally, marital status was considered a binary variable with *married* including women reporting being currently married or living as married and *not married* including widowed, divorced, separated, and never married women.

#### 2.3.2 Socioeconomic characteristics

The socioeconomic factors included in our analysis were annual household income ((1) under $20,000; (2) $20,000 to $99,999; and (3) $100,000 and above) and highest educational attainment ((1) high school/GED or less; (2) some college or associate degree; (3) bachelor's degree; and (4) graduate degree).

#### 2.3.3 Heath characteristics

The health factors included in our analysis were smoking status, alcohol consumption, BMI, and number of first‐degree female relatives with breast cancer. Smoking status response categories were (1) never; (2) past; and (3) current smoker. Alcohol consumption was characterized by (1) current drinker or (2) not‐current drinker. The BMI categories were (1) underweight/normal weight (under 25 kg/m^2^); (2) overweight (25–29.9 kg/m^2^); and (3) obese (30 kg/m^2^ and above). Lastly, we used three levels for number of first‐degree female relatives with breast cancer: (1) one; (2) two; and (3) three or more. We considered first‐degree female relatives to be full‐ or half‐sisters, mothers, and daughters.

### 2.4 Statistical analysis

We conducted multinomial logistic regression using the Sister Study cohort baseline data to examine the associations between sociodemographic, socioeconomic, and health characteristics and timing of most recent breast cancer screening in non‐Hispanic/Latina White and Hispanic/Latina women with a family history of the disease. Our multinomial logistic regression models had timing of most recent breast cancer screening as the dependent variable, with recently screened as the reference group. The following independent variables were included: sociodemographic indicators (Model 1); socioeconomic indicators (Model 2); and health indicators (Model 3). All variables were retained in subsequent models. Sensitivity analyses were conducted in which women under 40 years of age were removed from the sample (*n* = 1764). Analyses were conducted using StataSE 16. All analytical files are available by reasonable request to the Sister Study.

## 3 RESULTS

### 3.1 Sample characteristics

Our sample consisted of 44,524 women. Of these participants, 92% were US‐born non‐Hispanic/Latina White, 2% were US‐born Hispanic/Latina, 2% were foreign‐born non‐Hispanic/Latina White, and 3% were foreign‐born Hispanic/Latina. The majority of women were 50 years of age or older (73%), married or living as married (77%), had an annual household income between $20,000 and $99,999 (61%), and had attained a bachelor's degree or higher (51%). Additionally, most women reported drinking alcohol (82%), never smoking (55%), and having one first‐degree female relative with breast cancer (73%). Most women in the sample (97%) received breast cancer screening in the past two years. See Table 1 for complete sample characteristics.

**TABLE 1:** Sample descriptive statistics stratified by screening history classification

Overall	Recently screened		Formerly screened		Never screened		Overall	
	*N*	%	*N*	%	*N*	%	*N*	%
	43,015	97	1102	2	407	1	44,524	100
Sociodemographic characteristics								
Birthplace and race/ethnicity								
US born								
Non‐Hispanic/Latina white	39,846	93	974	88	343	84	41,163	92
Hispanic/Latina	1019	2	47	4	19	5	1085	2
Foreign born								
Non‐Hispanic/Latina white	828	2	21	2	8	2	857	2
Hispanic/Latina	1322	3	60	5	37	9	1419	
Age								
<40	1545	4	80	7	139	34	1764	4
40–49	9952	23	342	31	138	34	10,432	23
≥50	31,518	73	680	62	130	32	32,328	73
Marital status								
Never married/widowed/divorced/separated	9760	23	393	36	111	27	10,264	23
Married/living as married	33,252	77	709	64	296	73	34,257	77
Missing	3		0		0		3	
Socioeconomic characteristics								
Income								
<$20,000	1724	4	136	13	48	12	1908	4
$20,000–$99,999	25,188	61	732	68	242	61	26,162	61
≥$100,000	14,359	35	201	19	108	27	14,668	34
Missing	1744		33		9		1786	
Educational attainment								
High school/GED or less	6736	16	241	22	67	16	7044	16
Some college or associate degree	14,348	33	420	38	141	35	14,909	33
Bachelor's degree	11,668	27	251	23	125	31	12,044	27
Graduate degree	10,261	24	189	17	74	18	10,524	24
Missing	2		1		0		3	
Health factors								
Smoking status								
Never smoked	23,866	55	558	51	247	61	24,671	55
Past smoker	15,862	37	356	32	99	24	16,317	37
Current smoker	3285	8	187	17	60	15	3532	8
Missing	2		1		1		4	
Current drinker								
No	7481	17	263	24	84	21	7828	18
Yes	35,527	83	839	76	323	79	36,689	82
Missing	7		0		0		7	
BMI								
<25	17,583	41	378	34	172	42	18,133	41
25–29.9	13,652	32	316	29	115	28	14,083	32
≥30	11,767	27	406	37	120	29	12,293	28
Missing	13		2		0		15	
No. 1st‐degree female relatives with BC								
One	31,424	73	886	80	355	87	32,665	73
Two	10,230	24	196	18	49	12	10,475	24
Three or more	1361	3	20	2	3	1	1384	3

Abbreviations: BC, breast cancer; BMI, body mass index.

### 3.2 Multivariable models

Model 1 (*N* = 44,521) included sociodemographic characteristics. This model found that compared to US‐born non‐Hispanic/Latina White women, foreign‐born Hispanic/Latina women had higher odds (odds ratio [OR] = 2.57, 95% confidence interval [CI] = 1.80–3.66) of having never been screened for breast cancer than of having been recently screened. Further, the odds of having been formerly screened for US‐born Hispanic/Latina women and for foreign‐born Hispanic/Latina women were 1.62 (95% CI = 1.20–2.19) and 1.66 (95% CI = 1.27–2.17), respectively, compared to US‐born White women.

Model 2 (*N* = 42,732) retained sociodemographic variables and added income and education. After controlling for socioeconomic indicators, the odds of having never been screened for breast cancer for foreign‐born Hispanic/Latina women was 1.71 (95% CI = 1.16–2.52) compared to US‐born non‐Hispanic/Latina White women. Additionally, compared to recent screening, the odds of former screening in US‐born Hispanic/Latina women were 50% (95% CI = 1.10–2.03) higher than US‐born non‐Hispanic/Latina women. These findings indicate substantial confounding by socioeconomic indicators on the relationship between birthplace/race/ethnicity and breast cancer screening history. See Table 2 for complete results.

**TABLE 2:** Odds ratios and confidence intervals for associations between combined birthplace and race/ethnicity and timing of most recent screening with recently screened as reference (models 1–3), Sister Study, 2003–2009

	Model 1: Sociodemographic factors^a^ (*N* = 44,521)						Model 2: Socioeconomic factors^b^(*N* = 42,732)						Model 3: Health factors^c^(*N* = 42,709)					
	Formerly screened			Never screened			Formerly screened			Never screened			Formerly screened			Never screened		
		95% CI			95% CI			95% CI			95% CI			95% CI			95% CI	
	OR	Lower	Upper	OR	Lower	Upper	OR	Lower	Upper	OR	Lower	Upper	OR	Lower	Upper	OR	Lower	Upper
Birthplace and race/ethnicity																		
US born																		
Non‐Hispanic/Latina White	ref.			ref.			ref.			ref.			ref.			ref.		
Hispanic/Latina	**1.62**	**1.20**	**2.19**	1.42	0.88	2.28	**1.50**	**1.10**	**2.03**	1.36	0.84	2.20	**1.47**	**1.08**	**2.00**	1.33	0.82	2.15
Foreign born																		
Non‐Hispanic/Latina White	1.02	0.66	1.58	1.05	0.52	2.14	1.03	0.66	1.62	1.12	0.55	2.28	1.06	0.68	1.66	1.13	0.55	2.32
Hispanic/Latina	**1.66**	**1.27**	**2.17**	**2.57**	**1.80**	**3.66**	1.14	0.86	1.51	**1.71**	**1.16**	**2.52**	1.15	0.87	1.54	**1.63**	**1.10**	**2.41**

*Note*: OR, odds ratio, CI, confidence interval. BMI (body mass index), and number of 1st‐degree female relatives who have been diagnosed with breast cancer. Bold shows *p* < 0.05.

^a^ Adjusted for age and marital status.

^b^ Adjusted for age, marital status, income, and education.

^c^ Adjusted for age, marital status, income, education, alcohol consumption, smoking status.

Model 3 (*N* = 42,709) incorporated health characteristics—smoking status, alcohol consumption, BMI, and number of first‐degree female relatives with breast cancer—and retained sociodemographic and socioeconomic controls from the previous models. Model 3, the most comprehensive model, was selected for interpretation.

### 3.3 Comprehensive model

Compared to US‐born non‐Hispanic/Latina White women, foreign‐born Hispanic/Latina women had higher odds of having never received breast cancer screening, relative to receiving a recent screening (OR = 1.63, 95% CI = 1.10–2.41). Similarly, US‐born Hispanic/Latina women were more likely than US‐born non‐Hispanic/Latina White women to receive former screening, relative to recent screening (OR = 1.47, 95% CI = 1.08–2.00). The differences in odds ratios for combined birthplace and race/ethnicity between models 2 and 3 suggest limited confounding from the health indicators. See Table 2 for complete results.

Removing women under 40 from the sample population did not change our findings. Results from the sensitivity analyses are available online as supplemental information.

## 4 DISCUSSION

Our analysis found distinct birthplace and racial/ethnic differences in screening backgrounds among the women in our study, while controlling for sociodemographic, socioeconomic, and health factors. US‐born Hispanic/Latina women were more likely than US‐born non‐Hispanic/Latina White women to have had their most recent breast cancer screening over 2 years ago. Furthermore, foreign‐born Hispanic/Latina women were more likely to have never received a breast cancer screening than US‐born non‐Hispanic/Latina White women. Despite the relatively low risk of breast cancer among Hispanic/Latina women in the United States, these findings underscore the vulnerability of immigrants and ethnic minorities to late‐stage detection of malignancies.

While socioeconomic factors appeared to confound the relationship between birthplace/race/ethnicity and screening history, birthplace and race/ethnicity were significantly associated with breast cancer screening utilization even after controlling for income and education. Compared to US‐born non‐Hispanic/Latina White women, US‐born Hispanic/Latina women were less likely to have received their most recent screening in the past 2 years and foreign‐born Hispanic/Latina women were less likely to have ever received breast cancer screening in their lifetime. These findings are consistent with documented disparities in access to screening among minority ethnic groups and foreign‐born women.^11^, ^23^ Goel *et al*.^24^ additionally found foreign birth to be associated with lower rates of cancer screening among Hispanics/Latinos. Although we did not analyze the effects of birthplace on screening in a Hispanic/Latina subpopulation, we found distinct screening disparities in foreign‐ and US‐born Hispanic/Latina women when compared to US‐born non‐Hispanic/Latina White women. Furthermore, socioeconomic characteristics, such as income and education, have been identified as risk factors influencing stage at diagnosis and survival in breast cancer patients.^25^ Meissner *et al*.^26^ previously identified cost as one of the leading barriers to mammography. Several studies have found associations between low socioeconomic status and underutilization of screening services in Hispanic/Latina women.^13^, ^27^ As Hispanics/Latinos in the United States have lower income and educational attainment than Whites on average,^28^ they are at greater risk of underutilizing preventive health services. Consistent with these findings, we found income and education to be significant confounders in the relationship between birthplace/race/ethnicity and screening. Foreign‐born Hispanic/Latina women possess intersecting identities and face numerous structural inequities. This intersectionality amounts to significant barriers to accessing preventive health care in the United States.

Our analysis did not indicate significant confounding from our health indicators—smoking status, alcohol consumption, BMI, and breast cancer family history—on the association between birthplace/race/ethnicity and screening history. However, past research has revealed potential “spillovers”^8^ between preventive health behaviors and screening tendencies. Bostean *et al*.^8^ found a positive association between normal BMI and breast cancer screening among White women. On the other hand, their study found mixed associations by race between alcohol consumption and screening. Lastly, they only found never smoking to be positively associated with screening among Black women. More research is needed on the associations and “spillovers” between health behaviors. This is of particular importance as preventive health behaviors of foreign‐born women can be impacted by the disruptive process of migration and further affected by acculturation once in the United States.^11^, ^16^ Previous research has similarly found associations between family history and screening habits. For instance, several studies have reported that women with first‐degree family history of breast cancer are more likely to be up to date with mammography screening guidelines than those with no family history.^8^, ^29^ This could be due to higher perceived risk of individual diagnosis, or higher perceived effectiveness of screening, among women with multiple close relatives with the disease. Nevertheless, women are confronted with contradictory breast cancer screening recommendations from sources such as the ACS and USPSTF which can lead to confusion over when to initiate and how often to receive screening.

Self‐reported family history of cancer is used by healthcare providers to make screening recommendations. As such, persons who may not have knowledge of, who do not report, or who are not asked about family breast cancer history are vulnerable to underutilization of preventive and screening services that could otherwise reduce mortality and poor health outcomes. Racial and ethnic minorities, including Hispanics/Latinos, as well as immigrants, are less likely than non‐Hispanic/Latino White individuals and the US‐born to report family history of cancer.^30^ These differences, if not considered by health professionals, can widen the disparity between who receives breast cancer screening and genetic testing. The Sister Study cohort is a unique population because all participants, at minimum, have knowledge of a sister with a previous breast cancer diagnosis. Despite this, we observed underutilization of breast cancer screening among US‐ and foreign‐born Hispanic/Latina women. Healthy People 2020 reported an increase in screening rates among Hispanic/Latina women in the United States between 2008 and 2015.^31^ However, despite narrowing gaps among average‐risk women, disparities in use of preventative services among high‐risk minority women must continue to be addressed.

Immigrant and ethnic minority populations are at higher risk than US‐born non‐Hispanic/Latino White individuals of deteriorating health due to factors associated with migration, acculturation, and structural inequities. Our findings show that Hispanic/Latina women, particularly those in a higher‐than‐average‐risk population, would benefit from tailored public health efforts to improve utilization of breast cancer screening services. To address the underutilization of breast cancer screening services among Hispanic/Latina women, interventions must bear in mind the unique barriers to screening for the foreign‐ and US‐born. Messaging campaigns and interventions aimed at increasing awareness of publicly available screening services should be culturally and linguistically appropriate and should promote intrafamilial conversations about family health history. Breast cancer family history contributes to personal risk and recommendations for preventative services made by physicians. Educational programs for health care providers should include training on the specific risk factors faced by minorities and immigrants.

### 4.1 Limitations

Our study is not without limitations. We conducted a cross‐sectional analysis which does not capture the temporal nature of breast cancer screening history. As we did not have data on the timing of the most recent MRI or ultrasound received by participants, we were limited by an incomplete picture of their screening backgrounds. Additionally, breast cancer screening history was self‐reported which could lead to overreporting of recent screening. The Sister Study cohort only includes women with a sister who has been diagnosed with breast cancer. Therefore, our findings are not generalizable to all US women. Lastly, by not differentiating between Hispanic/Latina subpopulations, our results are generalizations about a highly heterogeneous population. However, ours is the first study to investigate place of birth and racial/ethnic screening disparities in the Sister Study cohort and is an important contribution to the literature on breast cancer screening disparities.

### 4.2 Conclusion

Hispanic/Latina women in the United States carry an unequal burden of late‐stage breast cancer diagnosis. Our findings shed new light on the impact of ethnicity and birthplace on screening among a higher‐than‐average‐risk population. Specifically, these results highlight the importance of including birthplace when conducting analyses on minority groups. The observed disparities in our analysis further verify the vulnerability of Hispanic/Latina women to falling through the cracks of our preventive healthcare system. As such, it is of the upmost importance to eliminate disparities in who receives preventive healthcare services in the United States. Additionally, healthcare providers should be aware of potential underreporting of family history among minority and foreign‐born patients so as not to perpetuate inequities.

## CONFLICT OF INTEREST

The authors declare no potential conflicts of interest.

## AUTHOR CONTRIBUTIONS

CJT conducted data analysis, interpreted results, and drafted the manuscript. FW conceptualized and designed the analysis, acquired the data, and supervised manuscript preparation. FAMI helped conceptualized the analysis and prepare and interpret data. KMO validated the data analysis and edited the manuscript. CJT, KMO, and DSP substantially modified the submitted version of the manuscript. All authors read and approved the final version of the manuscript.

## ETHICS APPROVAL

The Sister Study was approved by the Institutional Review Board (IRB) of the National Institute of Environmental Health Sciences and the Copernicus Group IRB.

## Supporting information


Table S1

## Data Availability

The data that support the findings of this study are restricted but available via application to the Sister Study website (https://sisterstudy.niehs.nih.gov).

## References

[1] American Cancer Society . Cancer Facts & Figures 2021. American Cancer Society, Inc.; 2021.

[2] American Cancer Society . Cancer Facts & Figures for Hispanics/Latinos 2018–2020. American Cancer Society, Inc; 2018.

[3] American Cancer Society . Breast Cancer Facts & Figures 2019–2020. American Cancer Society, Inc.; 2019.

[4] Collaborative Group on Hormonal Factors in Breast Cancer . Familial breast cancer: collaborative reanalysis of individual data from 52 epidemiological studies including 58 209 women with breast cancer and 101 986 women without the disease. The Lancet. 2001;358(9291):1389‐1399. 10.1016/s0140-6736(01)06524-2 11705483

[5] Niehoff NM , Nichols HB , Zhao S , White AJ , Sandler DP . Adult Physical Activity and Breast Cancer Risk in Women with a Family History of Breast Cancer. Cancer Epidemiol Biomarkers Prev. 2019;28(1):51‐58. 30333218 10.1158/1055-9965.EPI-18-0674 PMC6325010

[6] Anderson AS , Caswell S , Macleod M , et al. Health Behaviors and their relationship with disease control in people attending genetic clinics with a family history of breast or colorectal cancer. J Genet Couns. 2017;26(1):40‐51. 27312973 10.1007/s10897-016-9977-2 PMC5258810

[7] Amadou A , Hainaut P , Romieu I . Role of obesity in the risk of breast cancer: Lessons from anthropometry. J Oncol. 2013;2013:19‐19. 10.1155/2013/906495 PMC3575614 23431300

[8] Bostean G , Crespi CM , McCarthy WJ . Associations among family history of cancer, cancer screening and lifestyle behaviors: a population‐based study. Cancer Causes Control. 2013;24(8):1491‐1503. 23681471 10.1007/s10552-013-0226-9 PMC3871905

[9] Wernli KJ , Arao RF , Hubbard RA , et al. Change in breast cancer screening intervals since the 2009 USPSTF guideline. J Womens Health (Larchmt). 2017;26(8):820‐827. 28177856 10.1089/jwh.2016.6076 PMC5576213

[10] Budiman A , Tamir C , Mora L , Noe‐Bustamante L . Facts on US immigrants, 2018. 2020 (accessed February 2 2021).

[11] Ghirimoldi F , Sanchez‐Soto G . Immigrant assimilation and profiles of breast cancer screening behaviors among U.S. immigrant women. Health Care Women Int. 2020;42:1‐22. 10.1080/07399332.2020.1797034 32779966

[12] Singh G , Rodriguez‐Lainz A , Kogan M . Immigrant health inequalities in the United States: use of eight major national data systems. Sci World J. 2013;2013:1‐21. 10.1155/2013/512313 PMC3826317 24288488

[13] Rodriguez M , Ward L , Perez‐Stable E . Breast and cervical cancer screening: Impact of health insurance status, ethnicity and nativity of Latinas. Ann Fam Med. 2005;3(3):235‐241. 15928227 10.1370/afm.291 PMC1466881

[14] Abraído‐Lanza AF , Chao MT , Flórez KR . Do healthy behaviors decline with greater acculturation? Implications for the Latino mortality paradox. Soc Sci Med. 2005;61(6):1243‐1255. 15970234 10.1016/j.socscimed.2005.01.016 PMC3587355

[15] Abraído‐Lanza AF , Armbrister AN , Flórez KR , Aguirre AN . Toward a theory‐driven model of acculturation in public health research. Am J Public Health. 2006;96(8):1342‐1346. 16809597 10.2105/AJPH.2005.064980 PMC1522104

[16] Joshi S , Jatrana S , Paradies Y , Priest N . Differences in health behaviours between immigrant and non‐immigrant groups: a protocol for a systematic review. Syst Rev. 2014;3:6. 10.1186/2046-4053-3-61 PMC4062644 24915754

[17] Clarke TC , Endeshaw M , Duran D , Saraiya M . Breast cancer screening among women by nativity, birthplace, and length of time in the United States. Natl Health Stat Report. 2019;129:1‐15. 31751203

[18] Healthy People . Women receiving a mammogram within past 2 years (age‐adjusted, percent, 50–74 years). https://www.healthypeople.gov/2020/data/Chart/4055 (accessed October 28 2021).

[19] Stern MC , Fejerman L , Das R , et al. Variability in cancer risk and outcomes within us Latinos by national origin and genetic ancestry. Curr Epidemiol Rep. 2016;3:181‐190. 27547694 10.1007/s40471-016-0083-7 PMC4978756

[20] Pinheiro P , Sherman R , Trapido E , et al. Cancer Incidence in First Generation US Hispanics: Cubans, Mexicans, Puerto Ricans, and New Latinos. Cancer Epidemiol Biomarkers Prev. 2009;18(8):2162‐2169. 19661072 10.1158/1055-9965.EPI-09-0329

[21] Sandler DP , Hodgson ME , Deming‐Halverson SL , et al. The Sister Study Cohort: Baseline Methods and Participant Characteristics. Environ Health Perspect. 2017;125(12):127003. 29373861 10.1289/EHP1923 PMC5963586

[22] Data Requests. https://sisterstudy.niehs.nih.gov/English/data‐requests.htm.

[23] Wu TY , West B , Chen YW , Hergert C . Health beliefs and practices related to breast cancer screening in Filipino, Chinese and Asian‐Indian women. Cancer Detect Prev. 2006;30(1):58‐66. 16458452 10.1016/j.cdp.2005.06.013

[24] Goel MS , Wee CC , McCarthy EP , Davis RB , Ngo‐Metzger Q , Phillips RS . Racial and ethnic disparities in cancer screening – The importance of foreign birth as a barrier to care. J Gen Intern Med. 2003;18(12):1028‐1035. 14687262 10.1111/j.1525-1497.2003.20807.x PMC1494963

[25] Coughlin SS . Social determinants of breast cancer risk, stage, and survival. Breast Cancer Res Treat. 2019;177(3):537‐548. 31270761 10.1007/s10549-019-05340-7

[26] Meissner HI , Klabunde CN , Breen N , Zapka JM . Breast and colorectal cancer screening U.S. primary care physicians' reports of barriers. Am J Prev Med. 2012;43(6):584‐589. 23159253 10.1016/j.amepre.2012.08.016

[27] Jerome‐D'Emilia B . A systematic review of barriers and facilitators to mammography in Hispanic women. J Transcult Nurs. 2015;26(1):73‐82. 24797255 10.1177/1043659614530761

[28] Williams DR , Priest N , Anderson NB . Understanding associations among race, socioeconomic status, and health: Patterns and prospects. Health Psychol. 2016;35(4):407‐411. 27018733 10.1037/hea0000242 PMC4817358

[29] Townsend JS , Steele CB , Richardson LC , Stewart SL . Health behaviors and cancer screening among Californians with a family history of cancer. Genet Med. 2013;15(3):212‐221. 23018750 10.1038/gim.2012.118 PMC4394991

[30] Orom H , Coté ML , González HM , Underwood W , Schwartz AG . Family history of cancer: is it an accurate indicator of cancer risk in the immigrant population? Cancer. 2008;112(2):399‐406. 18072272 10.1002/cncr.23173

[31] Henley SJ , Thomas CC , Lewis DR , et al. Annual report to the nation on the status of cancer, part II: Progress toward Healthy People 2020 objectives for 4 common cancers. Cancer. 2020;126(10):2250‐2266. 32162329 10.1002/cncr.32801 PMC7223723

