# Supplementary figures and images for: Breast cancer screening among Hispanic and non‐Hispanic White women by birthplace in the Sister Study
Source: Cancer Med. 2022 Feb 12;11(8):1913–22. doi: 10.1002/cam4.4563 (PMC9041086; doi:10.1002/cam4.4563)

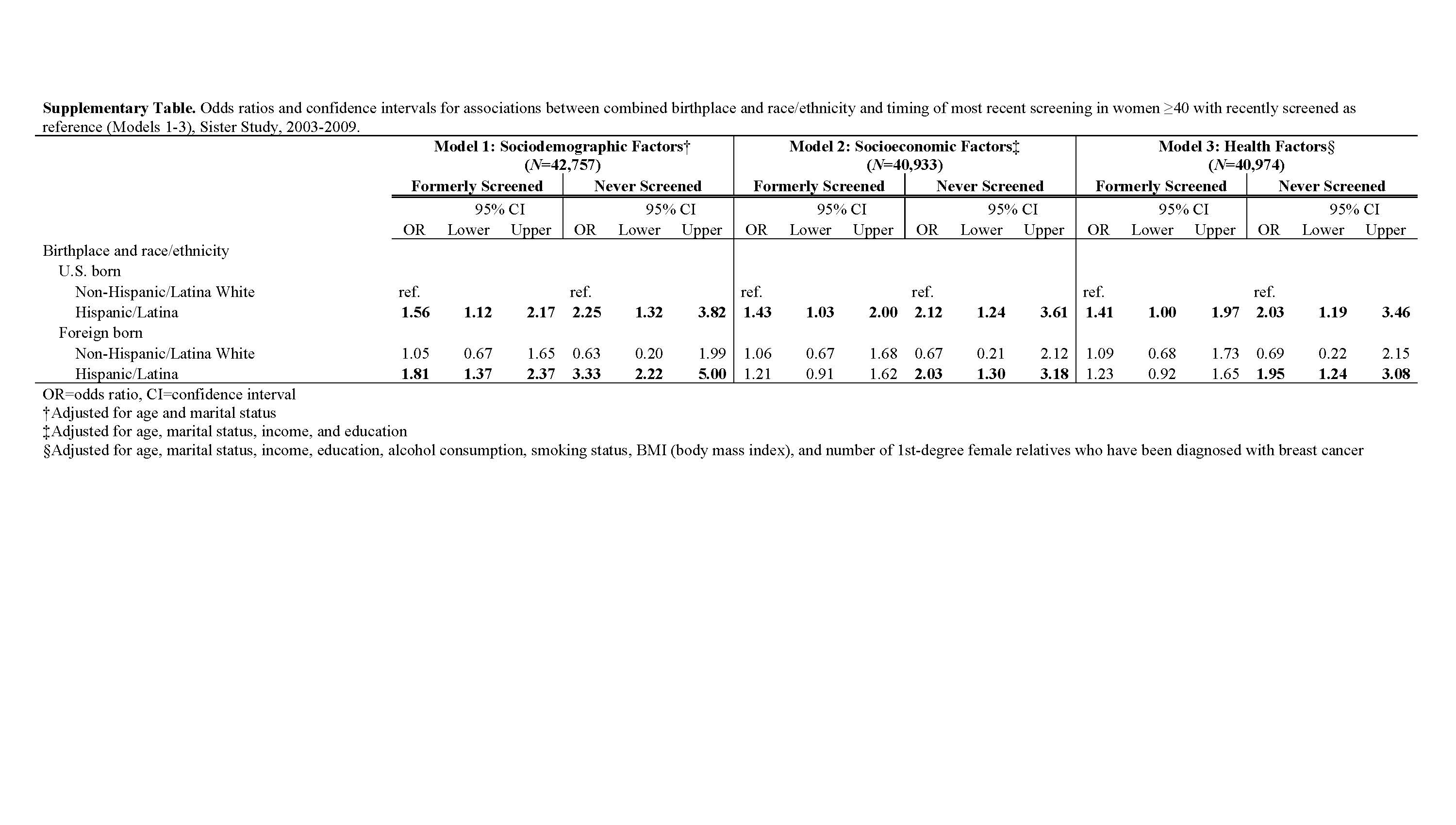

Supplement: Supplementary file 1 — Table S1 [file CAM4-11-1913-s001.jpg]
